# Selective Bacterial Colonization of the Murine Larynx in a Gnotobiotic Model

**DOI:** 10.3389/fmicb.2020.594617

**Published:** 2020-11-04

**Authors:** Ran An, Madhu Gowda, Federico E. Rey, Susan L. Thibeault

**Affiliations:** ^1^Department of Surgery, School of Medicine and Public Health, University of Wisconsin–Madison, Madison, WI, United States; ^2^Department of Bacteriology, University of Wisconsin–Madison, Madison, WI, United States

**Keywords:** gnotobiotic, laryngeal mucosa, microbiota, upper airway, colonization

## Abstract

The larynx is a mucosal organ situated between the respiratory and gastrointestinal tracts. Little is known about microbial contributions to laryngeal epithelial health and pathogenesis. Developing a gnotobiotic laryngeal model will introduce new avenues for targeted explorations of microbes in laryngeal mucosal biology, allowing for enhanced understanding of host–microbe interaction in the upper airway. In this study, we first assessed the potential of using gut microbiota as a source to establish laryngeal microbiota in germ-free mice. Results demonstrated the selective nature of the upper airway and provided evidence that gut bacteria can assemble into communities that resemble the commensal resident bacteria occurring in the larynx of conventionally-raised animals phylogenetically and functionally. Then, we confirmed the reproducibility of laryngeal colonization through comparison of laryngeal microbiota in the larynx along with neighboring regions (base of tongue, esophagus, and trachea) between conventionally-raised and germ-free mice that conventionalized with cecal microbiota. Despite taxonomic differences, the established laryngeal microbiota from cecal content exhibited similarity to commensal resident microbiota in diversity within/between communities and predicted metagenomic functions. Our data also suggests little difference in bacterial distribution across the larynx and its surrounding regions and that cell motility and the ability to degrade xenobiotics is critical for bacteria colonizing upper airway. Successful colonization of laryngeal and oropharyngeal regions with gut microbiota in our study will greatly facilitate the investigation of potential localized inflammatory responses within host tissues that contribute to the disorders of essential laryngeal functions. Utilizing said gnotobiotic model to conduct future studies will allow for novel insights into direct microbial contributions to laryngeal epithelial health and pathogenesis.

## Introduction

The use of gnotobiotic animal models, where microbial presence within an animal host can be controlled, has been fundamental to our current understanding for host–microbial relationships in various organ systems (Bibiloni, 2012; Williams, 2014). Gnotobiotic models have allowed for direct manipulation of microbial communities to evaluate the effects of specific members among local host regions along with systemic host immunology (Martín et al., 2016). This, coupled with recent advances in sequencing technologies, has granted the ability to characterize full microbial communities and dissect specific microbial contributions to shed light on host health and disease states (Cho and Blaser, 2012; Crouzet et al., 2013; Button et al., 2016; Richmond et al., 2016).

A large portion of clinically oriented microbiome work has focused on the gut and respiratory systems and respective disease states within these organs (Pimentel et al., 2013; Eun et al., 2014; Major and Spiller, 2014). Within the gut and respiratory tract, microbial contribution to inflammatory conditions such as inflammatory bowel syndrome (IBS) and Crohn’s disease (CD), chronic obstructive pulmonary disease (COPD) and cystic fibrosis, respectively have been examined (Dalal and Chang, 2014). However, a paucity of research exists specific to the larynx. The larynx lies at the intersection between these two well-investigated regions, with the vocal folds serving as a valving mechanism for voice production and airway protection during swallowing (Thibeault et al., 2009). Due to its locality, the larynx is frequently exposed to external substances originating from the gut or respiratory systems such as airborne particulates and refluxate (Thibeault et al., 2009). Despite this cross-over exposure from adjacent systems, the larynx appears to harbor unique microbiota (Jetté et al., 2016; Hanshew et al., 2017).

Microbial profiles in the larynx have been characterized in healthy and diseased populations with laryngeal pathologies spanning from reflux, chronic laryngitis, benign vocal fold lesions, and carcinoma (Gong et al., 2017; Hanshew et al., 2017). Despite the fact that non-uniform standards have been used in the evaluation of healthy controls in other studies, *Prevotella*, *Streptococcus*, and *Veillonella* have been found as abundant genera in all respiratory sites except anterior nares (Walker et al., 2014; Hanshew et al., 2017). The larynx microbiota from laryngeal carcinoma patients differs from that in benign vocal fold polyps (Gong et al., 2013). Interestingly, multiple lines of evidences suggest an association between *Streptococcus* and benign vocal fold lesions (Hanshew et al., 2014; Jetté et al., 2016). Further work exploring the effects of inflammatory irritants, specifically smoking and reflux, on the laryngeal microbiome revealed no microbial shifts associated with reflux status; however, reduced microbial diversity and differences in community structure was found in smokers compared with non-smokers. Further, smokers demonstrated differences in *Streptococcus*, unclassified *Comamonadaceae*, *Cloacibacterium*, and *Helicobacter* representation (Jetté et al., 2016). As such, current literature, though limited, suggests a microbial component in inflammatory disease pathogenesis. However, there has been no work investigating the direct effects of pathogens in disease progression.

Since our knowledge of the laryngeal microbiome and its association with host immunity is currently in its infancy, establishing an animal model within which microbial effects can be studied is vital for the understanding of the immunological host–microbe interrelationships contributing to vocal fold health and disease. Further, microbial characterization of neighboring regions within said gnotobiotic model would allow for the detection of region-specific communities and provide a comprehensive understanding of the cross section between the upper airway and gastrointestinal tract. While there has been one study defining pathological differences in the laryngeal epithelium of gnotobiotic rats (Lewis, 1982), there has been no standardized protocol to inoculate the laryngeal region for colonization within a gnotobiotic host. Inoculation of germ-free (GF) animals have historically consisted of transnasal, oral, or intragastric methods depending on the targeted organ system of study (Mebus and Underdahl, 1977; Berg and Garlington, 1979; Francis et al., 1986; Turnbaugh et al., 2009). Specifically, in the mouse model, orogastric or intragastric methods were employed to colonize mice with gut-associated pathogens (i.e., *Escherichia coli*, *Clostridium difficile*) or to inoculate GF mice with human fecal microbiota to establish humanized models resulting in successful transfer of target microbial members to the gnotobiotic specimens (Francis et al., 1986; Turnbaugh et al., 2009). This study examines gnotobiotic approaches for modeling host–microbe interactions in the larynx. We hypothesize that the unique characteristics of the larynx make this organ a selective environment for microbial colonization. We inoculated GF mice with larynx or gut communities and compared the engrafted microbiomes in the larynx and its neighboring regions with those from conventionally-raised (ConvR) mice. Our results show that microbes present in feces can assemble into communities that resemble phylogenetically and functionally, commensal resident bacteria occurring in ConvR animals, underscoring the selective nature of the upper airway.

## Materials and Methods

### Sample Collection

This study was completed in accordance with approved protocol (M005669) from the Animal Care and Use Committee at the University of Wisconsin–Madison.

First, two groups of GF male mice of 12-week old were oral-gavaged respectively with fecal slurry or laryngeal extract. Fecal inoculum was prepared from fecal samples collected from three ConvR mice raised in UW-Madison BRMS Mouse Breeding Core and resuspended in 3 mL of MegaMedia (Goodman et al., 2011) in hungate tubes, whereas laryngeal inoculum was prepared from three larynges excised with sterile surgical tools from ConvR animals in a biosafety cabinet, pooled, homogenized, and resuspended in the same media. Aliquots of the fecal and laryngeal inocula were preserved at −80°C for further analysis. Preparation of inocula, mouse colonization, and sample collection and processing were described in details in the following cecal microbiota transplantation experiment. All GF and colonized mice were housed at the UW-Madison gnotobiotic mice facility, provided with autoclavable mouse breeder diet Labdiet 5021 (Purina, St. Louis, MO, United States) and sterilized reverse osmosis water.

In a follow-up experiment, five GF male C57BL6 mice were colonized with cecal microbiota. To create the cecal inoculum, fresh cecal contents were collected from a ConvR C57BL6 mouse raised in the gnotobiotic mouse facility and immediately transferred into an anaerobic chamber for resuspension into 3 mL of MegaMedia in hungate tubes. Each mouse received 200 μl of inoculum via oral gavage at 12 weeks of age. To allow enough time for assembly and stabilization of microbial communities, conventionalized mice (ConvD) were maintained in the gnotobiotic facility for 4 weeks, then sacrificed via cervical dislocation and decapitation at 16 weeks of age. Base of tongue (BOT), esophagus (ESO), larynx (LAR), and trachea (TRA) regions were surgically excised under microscopy and stored at −80°C until further processing. BOT was collected along the superficial layer of the posterior portion of the tongue mass. Extraneous strap musculature surrounding the larynx was removed, and the larynx-trachea-esophagus complex was isolated. The anterior esophageal wall was separated from the posterior TRA, and ESO samples were dissected from the laryngeal complex at the point of the upper esophageal sphincter. TRA samples were separated from LAR via separation immediately inferior to the cricoid cartilage. The entire length of TRA and ESO was collected spanning from the point of decapitation to the respective margins of dissection. The remaining larynx complex inclusive of thyroid cartilage and tissues within the laryngeal vestibule (false and true vocal folds) was collected to serve as the larynx sample. The same regions (BOT, ESO, LAR, TRA) were harvested from GF mice (*n* = 5) raised within the same gnotobiotic mice facility in addition to ConvR mice (*n* = 5) housed in the same gnotobiotic mice facility to serve as comparative controls. The same diet and water as above were supplied to the mice in this experiment.

### Sample Processing

Tissues collected from sets of animals described above and preserved inocula were gently thawed at room temperature and DNA was extracted using the Epicentre MasterPure Complete DNA and RNA Purification Kit (Illumina, Madison, WI, United States) with modifications to the manufacturer’s protocol detailed as follows. Tissues were transferred to sterile screw top tubes containing 200 mg of 400 μm silica beads with 300 μl of Cell and Tissue Lysis solution added. Each sample was added with 100 μg of Proteinase K and then vortexed. Samples were incubated at 55°C for 1 h, with vortexing every 15 min. Samples were then transferred to 1.5 ml microcentrifuge tubes and vortexed at maximum speed for 10 min. 5 μg of RNaseA was added to the samples, mixed by vortexing, incubated at 37°C for 30 min, then placed on ice for 5 min. Subsequently, 175 μl of MPC Protein Precipitation Reagent was added to the sample. The remainder of the DNA extraction was executed per manufacturer’s protocol. Extracted DNA was resuspended in 42 μl of TE buffer, quantified using the Qubit^®^ Fluorometer (Invitrogen, San Diego, CA, United States) and stored at 4°C until further processing.

V3–V4 region of the 16S rRNA gene were amplified using AccuPrime^TM^ Hi Fidelity Taq (Thermo Fisher, Madison, WI, United States) in a 25 μl reaction containing 10 ng of DNA template, 200 μM 341F/785R primers. A no-template control (NTC), extraction negative control, and a positive control with *Helicobacter pylori* genomic DNA as template was included for each PCR run. PCR cycling conditions were as follows: one cycle of enzyme activation at 95°C for 3 min followed by 35 cycles of denaturation at 95°C for 30 s, annealing at 55°C for 30 s, and extension at 72°C for 30 s, and a final extension at 72°C for 5 min. Resulting PCR products were identified on 1% agarose gel. GF mouse samples, extraction negative controls, and NTC were confirmed to have no visible amplicon bands and were excluded from subsequent statistical analysis. The amplicon products generated from samples collected from colonized mice were cleaned using the PureLink^TM^ PCR Purification Kit (Thermo Fisher, Madison, WI, United States), quantified again using the Qubit^®^ Fluorometer, and stored at 4°C until index attachment. Index-tagging PCR was completed using AccuPrime^TM^ Hi Fidelity Taq in a 25 μl reaction containing 5 μl of amplicon PCR template, 400 μM of N7 and S5 Nextera XT barcoding primer sets as documented in the Illumina Miseq manufacturer’s protocol (Illumina, San Diego, CA, United States). PCR conditions were the same as the amplicon PCR run with the exception of running 12 cycles. Similarly, an NTC and a *Helicobacter pylori* positive control were included for each index-tagging PCR run. Resulting PCR products were identified on 1% gel, quantified with the Qubit^®^ Fluorometer, pooled into an equimolar library with 5% PhiX control DNA, and sequenced on an Illumina MiSeq platform with 250-bp paired-end sequencing chemistry (Illumina, San Diego, CA, United States) at UW-Madison Biotechnology Center.

Amplicon libraries for laryngeal tissues and inoculums for the first set of animals were sequenced separately in a 2 × 250 PE run, where V4 regions were amplified in the same PCR amplification system above using 515F/806R primers.

### Sequencing and Statistical Analysis

Demultiplexed sequences were processed, quality filtered, and analyzed with QIIME2^1^, a plugin-based microbiome analysis platform (Bolyen et al., 2019). Open source pipeline DADA2 was used to denoise sequencing reads with the q2-dada2 plugin for quality filtering and identification of *de novo* amplicon sequence variants (ASVs) (Callahan et al., 2016). This resulted in 1,021,385 high-quality sequences with an average of 29,029 sequences per sample for ConvR mice and 26,101 for ConvD mice (Suppementary Table 1). ASVs were aligned with mafft with q2-alignmnet plugin (Katoh and Standley, 2013). The q2-phylogeny plugin was used for phylogenetic reconstruction through FastTree (Price et al., 2010). Taxonomic classification was assigned using *classify-sklearn* against the Greengenes 13_8 99% reference sequences (DeSantis et al., 2006; McDonald et al., 2012; Bokulich et al., 2018). Microbial composition at each taxonomic level was defined using the *taxa-collapse* function in QIIME2. A lpha-diversity (Observed ASV richness, Pielou’s Evenness, and Shannon diversity) and beta-diversity (weighted and unweighted UniFrac) analyses were performed using q2-diversity plugin at a rarefaction depth of 3,000 sequences per sample. Three samples, including 1 LAR from ConvD, 1 TRA from each of ConvD and ConvR groups, were removed from subsequent analysis because they did not reach this sequencing depth. Subsequent processing and analysis were performed in R.

Microbial community difference between groups were evaluated through principal coordinates analysis (PCoA) and statistically examined by permutational analysis of variance (PERMANOVA) test. The core communities of ConvR and ConvD mice were determined using *feature-table core-feature* function in QIIME2, where they were defined as taxa observed in at least 90% of the total samples in each group. Indicator species analysis was performed by indicspecies package in R (De Cáceres et al., 2010). Shared ASVs between mouse groups and regions were determined and visualized by online tool^2^. Differential abundances between mouse groups were analyzed using linear discriminant analysis (LDA) effect size (LEfSe) (Segata et al., 2011). Bacterial taxa with LDA score > 2 and a *P*-value < 0.05 were considered significantly enriched.

PICRUSt2 was used to predict the functional potential of the engrafted laryngeal microbiomes originating from the different mouse groups and regions using 16S rRNA sequencing data (Douglas et al., 2019). Functional predictions were assigned to KEGG pathways (Kanehisa and Goto, 2000). PICRUSt2 results were normalized, and then analyzed using STAMP tool (Parks et al., 2014), where Welch’s *t*-Test was used to compare the difference in gene and pathway abundances between microbiome groups and regions at the level *P* = 0.05 (Effect size > 2) adjusted with Benjamini-Hochberg false discovery rate (FDR) method (Benjamini and Hochberg, 1995). Microbial phenotypes at the organism-level were also predicted using BugBase^3^ based on 16S rRNA gene sequence data in this study (Ward et al., 2017). DADA2 denoised ASVs were clustered against the GreenGenes 13_8 99% reference sequences. Kruskal–Wallis test was performed to test the statistical difference between groups (*P* = 0.05).

## Results

### Engraftment of Communities in the Larynx of Germ-Free Mice Inoculated With Fecal and Laryngeal Microbiota

**FIGURE 1:**
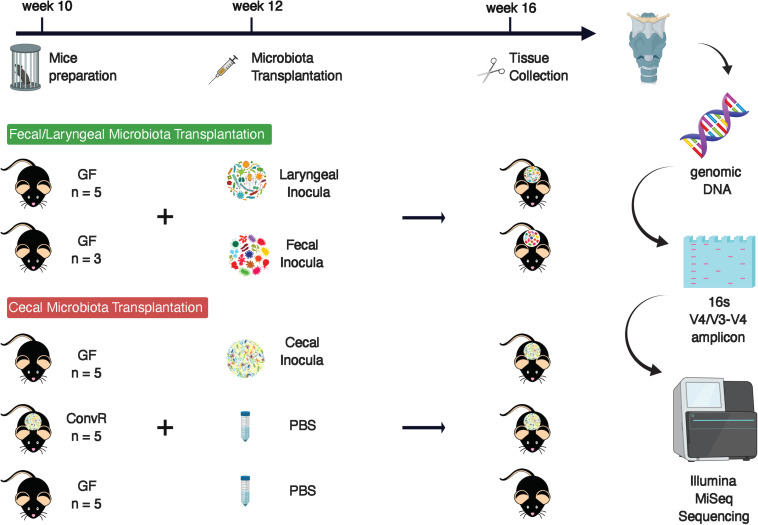
Experimental design.

**FIGURE 2:**
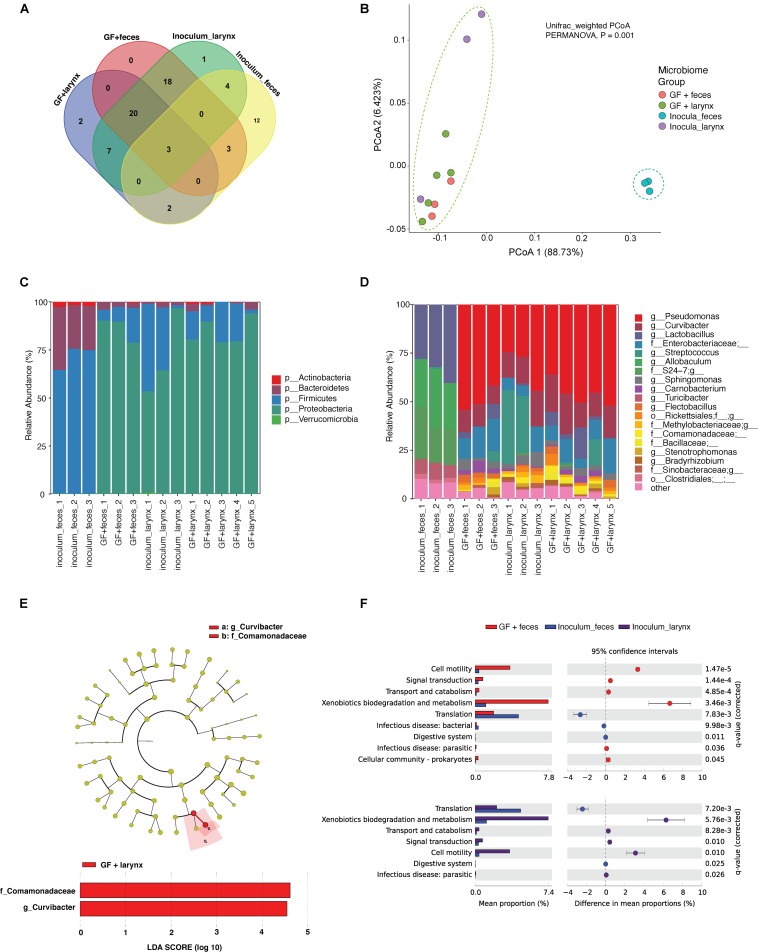
Characterization of microbiomes in the larynx of germ-free mice inoculated with fecal and laryngeal microbiota. **(A)** Amplicon sequence variants (ASVs) shared between transplanted mice and their inocula. ASVs present in only one sample and those with less than 0.1% of total abundance summed across all samples were removed prior to analysis. **(B)** Beta-diversity analysis showing difference in microbial community structure between transplanted mice and their respective inocula. Principal coordinates analysis (PCoA) plots of weighted UniFrac distances in transplanted mice and fecal/laryngeal inocula. Dotted contours indicate the groups obtained by comparisons with PERMANOVA (*P* = 0.001, *F* = 7.0). **(C,D)** Taxonomy compositions of microbiota at phylum and genus levels in the transplanted mice and their inocula. **(E)** Cladogram showing the significantly different taxa between transplanted mice that received laryngeal and fecal microbiota. Linear discriminate analysis effect size (LEfSe) was performed to determine the differentially abundant taxa between the two transplanted mouse groups at the level *P* = 0.05; the threshold of LDA score = 2.0. **(F)** Metagenomic functional prediction of KEGG pathways that are differentially represented in transplanted mice relative to their inocula. Welch’s *t*-test was performed to compare the difference in the pathways GF + feces, fecal inocula, and laryngeal incula at the level *P* = 0.05 (Effect size > 2) adjusted with Benjamini-Hochberg false discovery rate (FDR) method.

We performed a pilot experiment to investigate whether the larynx is a selective environment for bacterial colonization (Figure 1). Two groups of GF mice were inoculated by oral gavage with a laryngeal or fecal microbiota. Two weeks later we examined the composition of engrafted communities using 16S rRNA gene sequencing and compared to the respective inocula. After removing ASVs that were present in only one sample and those with less than 0.1% of total abundance summed across all samples, we identified 72 ASVs across the 14 samples comprising 3 fecal inocula, 3 laryngeal inocula, 3 gnotobiotic mice with fecal input microbiota, and 5 gnotobiotic mice with laryngeal input microbiota. Laryngeal inocula and gnotobiotic mouse groups shared 23 ASVs (Figure 2A) whereas, fecal inocula shared only 3 ASVs with others. PCoA of weighted UniFrac distances showed that the two gnotobiotic mouse groups with different input microbiota clustered together (Figure 2B), suggesting that despite the disparate microbiota input source, the communities assembled in larynx were similar and distinctly separated from fecal inocula. This was also reflected by the taxonomic profile of each microbiome group, where the gnotobiotic mice that received laryngeal and fecal microbiota, as well as laryngeal inocula, were dominated by Proteobacteria, whereas fecal inocula were dominated by Firmicutes and Bacteroidetes (Figures 2C,D). Several genera abundant in fecal inoculum, including *Lactobacillus*, *Allobaculum*, *Turicibacter*, and an unclassified genus from S24_7 family were significantly decreased/not present in the engrafted larynx microbiome; whereas, *Pseudomonas* and *Curvibacter* became dominant in the larynx of transplanted mice. Interestingly, the proportion of *Streptococcus* was considerably decreased in mice colonized with laryngeal input relative to their respective inocula. This could account for the separation between the laryngeal inocula and the engrafted larynx community observed in the PCoA. In comparing between the transplanted mouse groups, *Curvibacter* was the only genus differentially represented according to LEfSe analysis (LDA score > 2.0) (Figure 2E).

Metagenome functional profiles predicted by PICRUSt2 based on the 16S rRNA gene data suggested that microbes that colonized the larynx collectively encode for significantly distinct functions from the fecal inocula, regardless of input source (Supplementary Figure 1). Compared with fecal inocula, the relative abundance of five metabolic pathways were significantly increased in transplanted mice with fecal input microbiota (Effect size > 2), including pathways associated with cell motility and xenobiotics biodegradation/metabolism were enriched more than 5 folds in the transplanted mice according to a two-sided corrected Welch’s *t*-test at the level *P* = 0.05 (Effect size > 5) (Figure 2F Top). Similar enrichment was observed in the laryngeal inocula relative to fecal inocula (Figure 2F Bottom), indicating that the larynx, as a selective organ, shapes its own microbiome distinct from that in gut. This was also reflected by BugBase prediction at the organism level (Supplementary Figure 2). Laryngeal microbiota were predicted to harbor high abundance of bacteria that are gram-negative, aerobic, and potentially pathogenic, relative to fecal microbiota. The predicted relative abundance of bacteria with phenotypes, such as mobile genetic elements (MGE), biofilm-formation, and stress-tolerant potential, also tended to be higher in laryngeal microbiota, while the differences were marginally significant (*P* = 0.05, *F* = 36.5). Furthermore, no significant difference was observed between the two transplanted groups.

### Characterization of the Microbiome From the Larynx and Surrounding Tissues in a Gnotobiotic Model

**FIGURE 3:**
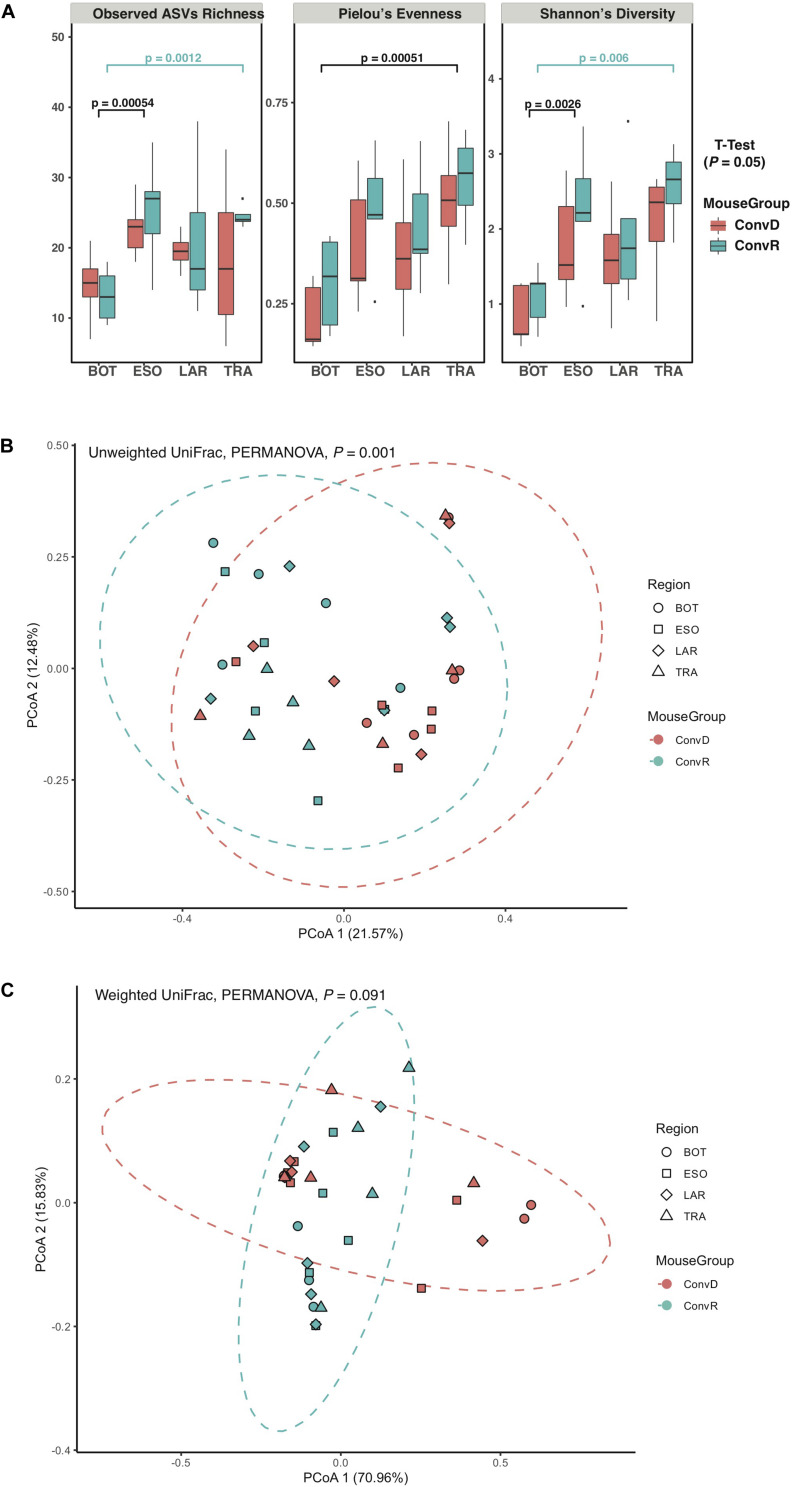
Alpha- and beta-diversity of microbiome from ConvD and ConvR mice. **(A)** Comparisons of alpha diversity indices between mouse groups and regions. **(B)** Beta diversity analysis showing difference in microbial community structure between ConvD and ConvR mice. PCoA of unweighted UniFrac distance. Dotted contours indicate the groups obtained by comparisons with PERMANOVA (*P* = 0.001, *F* = 7.0). **(C)** Beta diversity analysis showing difference in microbial community structure between ConvD and ConvR mice. PCoA of weighted UniFrac distances. Dotted contours indicate the groups obtained by comparisons with PERMANOVA (*P* = 0.091, *F* = 31.1).

The results of the initial study indicated that the larynx is a selective microbial environment and that fecal inoculum could be used as a substitute for laryngeal inoculum to establish similar microbial profiles in the larynx, as laryngeal inoculum does, allowing for analysis of microbiota in larynx and its surrounding regions using gnotobiotic mice colonized with exogenous microbiota. Specifically, we performed 16S rRNA gene sequencing for samples collected from ConvR and ConvD mice that received ConvR mouse cecal microbiota (*n* = 5/group) (Figure 1). We sampled the BOT, ESO, LAR, and TRA. Rarefaction curves showed that a sampling depth set as 3,000 was sufficient to detect all ASVs/features in each sample and to reliably describe the bacterial communities associated with each sample (Supplementary Figure 3). Regardless of the colonization status, the number of ASVs was on average lowest in the BOT relative to the other regions. Similarly, biodiversity (Shannon’s diversity) and evenness (Pielou’s evenness) were also lower in the BOT compared to the other regions, while there were no significant differences in most pairwise comparisons (Figure 3A). Furthermore, transplanted bacterial communities in each region maintained very similar richness, biodiversity and evenness relative to their ConvR counterparts.

Principal coordinates analysis of unweighted UniFrac distances of these samples revealed a weak, but statistically significant, clustering of bacterial communities, according to colonization group rather than region (*P* = 0.001, *F* = 7.0) (Figure 3B). On the other hand, the weighted UniFrac distances—a metric less sensitive to presence/absence of low-abundance ASVs, were not significantly different between groups (Figure 3C), implicating the presence of a large number of low-abundance ASVs in our data that could potentially impact the evaluation of difference in microbial community structure between ConvR and ConvD groups.

**FIGURE 4:**
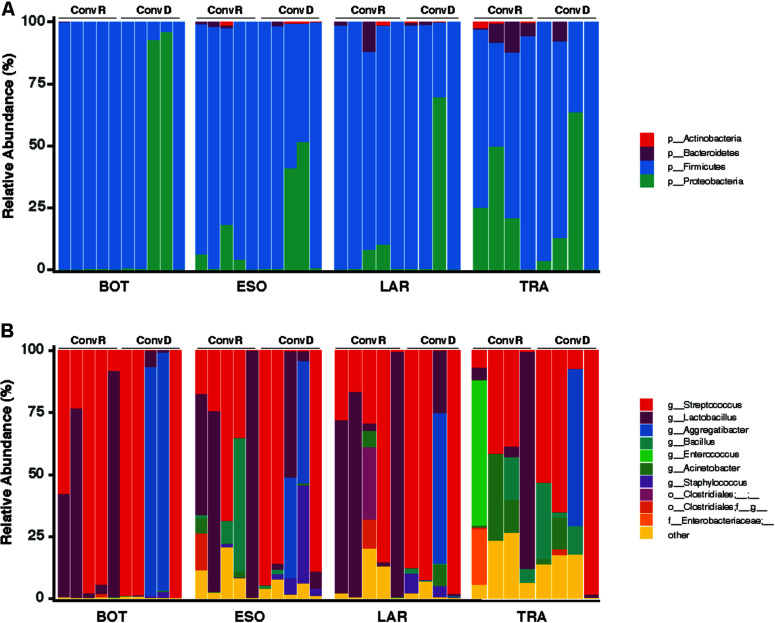
Taxonomy compositions of microbiota at phylum **(A)** and genus **(B)** level across the base of tongue (BOT), Esophagus (ESO), Larynx (LAR), Trachea (TRA) regions in ConvD and ConvR mice.

A total of 465 ASVs were identified across all samples, spanning 73 bacterial genera from 4 phyla— Firmicutes, Proteobacteria, Bacteroidetes, and Actinobacteria (Figure 4A). Firmicutes was the most abundant and the only phylum represented across all samples. The 10 most abundant ASVs, occupying 85% of the total ASV abundance identified in larynx and its surrounding regions belong to the Firmicutes and Proteobacteria (Table 1). *Streptococcus* and *Lactobacillus* were the most prominently represented genera among the Firmicutes, whereas *Aggregatibacter* and *Acinetobacter* were most prominently represented genera among the Proteobacteria (Figure 4B and Table 1).

**TABLE 1:** Top 10 abundant ASVs identified across 37 samples from ConvD and ConvR mice.

ASV	% of Total abundance	% of samples observed in	Phylum	Class	Order	Family	Genus	Species
ASV1	35	100	Firmicutes	Bacilli	Lactobacillales	Streptococcaceae	*Streptococcus*	Unclassified
ASV2	24	92	Firmicutes	Bacilli	Lactobacillales	Lactobacillaceae	*Lactobacillus*	Unclassified
ASV3	13	27	Proteobacteria	Gammaproteobacteria	Pasteurellales	Pasteurellaceae	*Aggregatibacter*	*pneumotropica*
ASV4	4	24	Firmicutes	Bacilli	Lactobacillales	Lactobacillaceae	*Lactobacillus*	Unclassified
ASV5	2	54	Firmicutes	Bacilli	Bacillales	Bacillaceae	*Bacillus*	*flexus*
ASV6	2	19	Proteobacteria	Gammaproteobacteria	Pasteurellales	Pasteurellaceae	*Aggregatibacter*	*pneumotropica*
ASV7	2	5	Firmicutes	Bacilli	Lactobacillales	Enterococcaceae	*Enterococcus*	*casseliflavus*
ASV8	1	24	Firmicutes	Bacilli	Bacillales	Staphylococcaceae	*Staphylococcus*	*sciuri*
ASV9	1	14	Firmicutes	Bacilli	Lactobacillales	Lactobacillaceae	*Lactobacillus*	Unclassified
ASV10	1	24	Proteobacteria	Gammaproteobacteria	Pseudomonadales	Moraxellaceae	*Acinetobacter*	Unclassified

### Variations in Microbial Community Composition Among Mouse Groups and Sampling Regions

ConvR and ConvD mouse groups shared 37 ASVs, which was only 8% of the total ASVs identified across all samples (Supplementary Figure 4). However, it accounts for over 70% of the total ASV abundance, encompassing 16 genera, including but not limited to the aforementioned most prominent genera (Table 1 and Supplementary Table 2). Each region shared very few ASVs between groups (Supplementary Table 3). The number and pattern of the shared ASVs among regions in ConvD mice were very similar to those in ConvR mice, which could possibly be an indication of the resemblance of commensal microbiota in ConvR mice (Supplementary Figure 5). *Streptococcus* was the only genus represented in every sample regardless of mouse group and sampling region. *Lactobacillus* was the second most prevalent genera, according to the “core microbiome” analysis, present in > 90% of samples from ConvR mice (Table 2). We also noticed core community members displayed variation across regions in ConvD mice, where *Staphylococcus* was more prevalent in BOT and *Bacillus* in LAR and TRA. Whereas, relatively low prevalence of *Lactobacillus* was observed in TRA in both ConvR and ConvD groups. Altogether, this evidence suggested the likely association of microbial community distribution with colonization status as well as sampling region. We used indicator species analysis to identify the genus that was most indicative of the colonization status and region (Table 3). Our results showed *Lactobacillus* as indicator for ConvR mice, and *Aggregatibacter*, *Staphylococcus*, and *Jeotgalicoccus* for ConvD mice (Table 3).

**TABLE 2:** Core microbiome across regions in ConvD and ConvR mice at the genus level.

Mouse group	Total	BOT	ESO	LAR	TRA
ConvR	*Lactobacillus*	*Lactobacillus*	*Lactobacillus*	*Lactobacillus*	*Streptococcus*
	*Streptococcus*	*Streptococcus*	*Streptococcus*	*Streptococcus*	
ConvD	*Streptococcus*	*Streptococcus*	*Lactobacillus*	*Streptococcus*	*Streptococcus*
		*Lactobacillus*	*Streptococcus*	*Lactobacillus*	*Bacillus*
		*Staphylococcus*		*Bacillus*	

*Taxa were listed in the order of high to low abundance.*

**TABLE 3:** Indicator species of the ConvR and ConvD mice.

Taxonomy (genus)	Group	Stat	*P*-value	Significant codes
*Aggregatibacter*	ConvD	0.424	0.0001	***
*Staphylococcus*		0.277	0.0015	**
*Jeotgalicoccus*		0.177	0.0489	*
*Lactobacillus*	ConvR	0.521	0.0007	***
*Cloacibacterium*	ConvR-TRA	0.606	0.0091	**
*Diaphorobacter*		0.536	0.0162	*
*Bacillus*	ConvD-TRA	0.613	0.0059	**

Significance codes: 0 ‘***’ 0.001 ‘**’ 0.01 ‘*’ 0.05.

**FIGURE 5:**
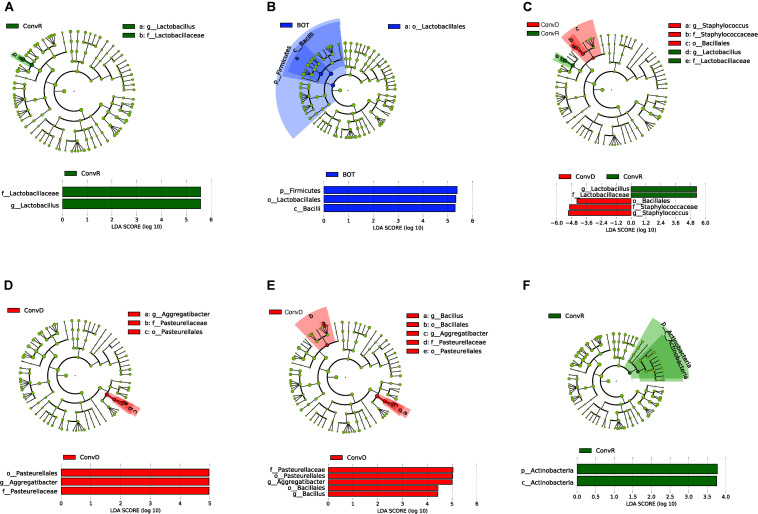
LEfSe analysis showing differentially abundant taxa between ConvD and ConvR mice in base of tongue (BOT), Esophagus (ESO), Larynx (LAR), Trachea (TRA). Threshold of LDA score = 2.0. Cladogram and horizontal histogram representation of **(A)** upper airway (all four regions) microbiota in ConvR vs. ConvD; **(B)** microbiota in BOT vs. other regions (ESO, TRA, and LAR) in ConvR; **(C)** BOT microbiota in ConvR vs. ConvD; **(D)** ESO microbiota in ConvR vs. ConvD; **(E)** LAR microbiota in ConvR vs. ConvD; **(F)** TRA microbiota in ConvR vs. ConvD.

We further examined the differentially abundant taxa, through LEfSe analysis, across mouse groups and regions (Figure 5). Compared between mouse groups, *Lactobacillus* was the only genus that was significantly enriched in ConvR mice (LDA scores > 5; Figure 5A). No differentially abundant taxa were found across regions in the colonized mice with cecal microbiota (Figure 5B). We observed differentially abundant taxa for each region between ConvR and ConvD animals. Specifically, the indicator genera for ConvD mice – *Staphylococcus* and *Aggregatibacter* – were represented at higher levels in the BOT and ESO of ConvD mice relative to ConvR mice, while *Aggregatibacter* and *Bacillus* were highly represented in the LAR of the same mouse group (LDA score > 4) (Figures 5C–E).

Metagenomic functional profiles predicted by PICRUSt2 revealed no KEGG pathways differentially represented between ConvR and ConvD mice (*P* = 0.05, Effect size > 2). Relative abundances of the predicted genes and pathways were relatively consistent across regions. No significant difference was observed either between regions in the same mouse group or between mouse groups for each region. This suggests the established laryngeal microbiota (ConvD) were similar to the commensal laryngeal microbiota (ConvR) not only phylogenetically also functionally.

## Discussion

In this study, we characterized microbial profiles of the larynx and its surrounding anatomical regions – BOT, ESO, and TRA– of gnotobiotic mice that received cecal microbiota of a ConvR mice using 16S rRNA gene sequencing. Initially, we assessed the potential of using gut microbiota as an input microbial source to create laryngeal microbiota in GF mice, that resembles commensal resident bacteria in ConvR mice. Our results suggest that gut microbiota, while different from laryngeal microbiota, contains taxa – most of them at low levels – can assemble into a community in the mouse larynx that largely resembles the community that is engrafted when GF mice are colonized with a larynx community (Figure 2). It also suggests that the mouse larynx is a selective environment that enriches for specific bacterial functions. This is very similar to mouse intestinal tract, a highly selective environment, but within the fundamental niche for microbes residing in a large number of environments (Seedorf et al., 2014). The laryngeal environment is aerobic and frequently exposed to external substances originating from the gut, respiratory system or air, such as airborne particulates and refluxate. This may impose selective pressures that favor the presence of genes/pathways associated with xenobiotics biodegradation and metabolism, which were found enriched in the larynx assemblages relative to the gut (Figure 2F). The increased abundances of aerobic bacteria and those with stress-tolerant and/or MGE – containing potentials are possibly a further indication of this hypothesis (Supplementary Figures 2A,G,I). Previous studies suggest stress-tolerant/MGE may have important roles in bacterial genome plasticity, host adaptation as well as degradation of xenobiotic compounds (Dealtry et al., 2014; Vigil-Stenman et al., 2017). Furthermore, the elevated abundances of biofilm-forming bacteria, which could be attributed partly, at least, to the enrichment of pathways involved in cell motility, including bacterial chemotaxis and flagellar assembly, is likely due to the distinct multilayered epithelial structure in larynx that is subjected to frequent external irritants and mechanical forces (Figure 2F and Supplementary Figure 2H) (Shrout et al., 2011). However, the functional data based on PICRUSt2 and BugBase cannot replace metagenomic, metatranscriptomic, or metabolomic studies. Experimental validation is required to prove our predictions.

The selectivity of larynx results in restructuring of the inoculum as they are introduced into a new environment (Figure 2). The similarity of communities assembled in the larynx of transplanted mice with fecal/laryngeal input microbiota provides a strong evidence according to our study. Firmicutes was the dominant phyla in the fecal inocula, while its dominance was replaced by Proteobacteria in the respective engrafted laryngeal microbiome. Several prominent genera in fecal inocula, such as *Allobaculum*, *Turicibacter*, and an unclassified genus from S24_7 family did not colonize while other genera did colonize, including but not limited to *Pseudomonas*, *Curvibacter*, and members of the *Enterobacteriaceae* in the engrafted larynx microbiome. It is likely that the metabolic capacities encoded by these genera allow them to flourish in the larynx. A much lower degree of community restructuring also occurred within the laryngeal inocula (Figure 2D). In this case the abundance of *Streptococcus* was moderately decreased in the transplanted mice with larynx microbiota, relative to their laryngeal inocula, which accompanied a significant increase in the abundance of *Lactobacillus*. This is possibly due to the previously observed antagonistic relationship between the two genera (Humphreys and McBain, 2019). The antagonistic interactions within and between bacterial species profoundly impact the outcome of competition (Humphreys and McBain, 2019). *Lactobacillus* is a common commensal resident in the gut, but not in the larynx. Its prevalence and dominance in larynx and its surrounding regions have been consistently observed in the transplanted mice with fecal and cecal input microbiota (Le Roy et al., 2015). However, it is difficult to delineate causative conclusions based on our current observation due to the limited sample size and the lack of functional analysis. Given that many *Lactobacillus* strains exhibit antibacterial activity against pathobionts, such as *Streptococcus*, and have been used to develop probiotics (Ruiz et al., 2013; Wang et al., 2016) we suggest the interplay between the two genera may play an important role in balancing their proportions in larynx. Altogether, this suggests that the transplanted gut microbial communities have undergone a complex reorganization to colonize the new ecological niche.

The selective nature of the larynx, combined with the presence of bacteria in fecal contents capable of colonizing this organ enable us to establish laryngeal communities in GF mice using gut contents as the inoculum. Therefore, we extended our analyses to compare the microbiota profiles in the larynx and its neighboring regions between ConvR and ConvD mice, that were colonized with cecal microbiota. It is important to note that the ConvR mice used in this study were from a different facility relative to the ones described in the first part of the paper, thus containing a very different gut and larynx microbiome. This may account for the ratio of Firmicutes and Proteobacteria abundances increased in ConvD mice compared with that of gnotobiotic mice colonized with fecal microbiota (Figures 2C, 4A). Taxonomic composition in input microbiota have profound impact on that of output microbiota (Seedorf et al., 2014). The proportions of major taxa of mouse gut microbiota vary considerably with individual mouse, cage, water, food, other specific details of husbandry, and even time (Rausch et al., 2016). Therefore, this work also underscores the importance of using the same inoculum when colonizing mice for a study. Larynx communities from ConvR and ConvD mice had no significant difference in alpha diversity for each region examined (Figure 3A). This is consistent with the previous study where the alpha diversities of gut microbiota were compared between multiple gnotobiotic mouse groups that received different input microbiota (Seedorf et al., 2014). However, unlike the resemblance of communities observed in our first experiment between the transplanted mice with fecal microbiota and laryngeal inocula used to work as ConvR mice, microbiomes of ConvR and ConvD mice were significantly different in taxonomy, including but not limited to, *Aggrigatibacter*, *Bacillus*, *Staphylococcus*, *Actinobacteria*, and *Lactobacillus*. This is mainly driven by the difference in the inocula used for the two experiments collected from the mice raised in different facilities. The impact of mouse facility on community composition and abundance were also reflected by the difference in microbiota between laryngeal inocula (fecal experiment) and ConvR mice (cecal experiment). We aimed to create laryngeal microbiota in GF mice that infinitely resembles the commensal resident bacteria in ConvR mice, using three inocula prepared from feces, cecum, and larynx. While our results support the establishment of the laryngeal microbiome via microbial introduction within an GF model, taxonomic differences compared to a natural acquisition of microbes from birth were present even with the most related/highly resembled microbiota source (laryngeal inocula) used for inoculation. Yet, our data suggests the established laryngeal microbiota resembles phylogenetically and functionally the commensal resident bacteria in the larynx, although differs taxonomically.

On the other hand, our data suggest that mouse fecal/cecal microbiome contains low levels of taxa that are either residents of the mouse larynx or that are closely related with taxa that can colonize the larynx. *Prevotella*, *Streptococcus* and *Veillonella* have been found as prominent community members in all respiratory sites except the anterior nares in healthy individuals (Hanshew et al., 2017). The high prevalence of *Streptococcus* found in our study compared well with these findings. However, the low abundance of *Prevotella* and *Veillonella* relative to previous reports could be due to the differences in the inoculum or host species that vary considerably with study. Besides, ConvD mice had a number of other taxa present at relatively low levels, including *Bifidobacterium*, *Enterobacter*, *Lachnospira*, *Sphingonomas*, *Cloacibacterium*, *Comamonodaceae*, etc., transplanted from cecal inocula (Supplementary Table 3), some of which, i.e., *Sphingonomas*, *Cloacibacterium*, *Comamonodaceae*, have been reported as commensal residents in human larynx and/or neighboring regions. The flourish of *Curvibacter*, a genus belongs to *Comamonodaceae* family, in the transplanted mice as well as the laryngeal inocula may suggest the similar selectivity of mouse larynx to that of human in establishing laryngeal microbiota, which further implies the resemblance in host selection pressure between mouse and human against xenomicrobiota. The increased abundance of *Curvibacter* in the transplanted mice with laryngeal input microbiota relative to the fecal input microbiota suggests its flourish may largely depends on its interplay with other members in the community. Moreover, the number of shared ASVs/taxa by ConvR and ConvD animals were comparatively lower than that of communities specific to each mouse group, especially in LAR (Supplementary Figure 5). This may implicate the unsuccessful colonization of some gut bacteria within the observation period after oral gavage (Supplementary Figures 2A,B). Adapting from the anaerobic gut environment to more oxygenated laryngeal and oropharyngeal environment is challenging to the vast majority of obligate anaerobes present in cecal matter used to create the inoculation slurry. It is speculated that the absence of certain anaerobic members presents competitive colonization opportunities within the ecological system; consequently, these gaps within the ecological structure would allow for the establishment of different microbial members unaffiliated with the inoculating community (Hibbing et al., 2010). The absence of aforementioned *Allobaculum*, *Turicibacter*, and an unclassified genus from S24_7 family is a strong evidence for this (Figure 2D).

Understanding the microbiomes in the surrounding regions of the larynx is as important to establish the laryngeal mouse model. Unified airway model suggests that the respiratory tract should be viewed as a single ecosystem. The different areas of the respiratory tract share many similar characteristics, and these shared traits likely extend to similar niche characteristics that support bacterial communities in similar mucosal surfaces (Hanshew et al., 2017). This accounts for the little or no differences in the abundances of successfully colonized taxa across regions in each mouse group (Figure 3 and Supplementary Table 2). *Bifidobacterium* was one of the successful colonizers specific to the larynx of ConvD mice. It is ubiquitously present in gastrointestinal tract, vagina, and mouth (Wong et al., 2020). Previous studies in gnotobiotic mice have shown it benefits host in various ways, including the induction of immune response, conferring resilience to chronic social defeat stress (CSDS), and alleviating constipation (Ménard et al., 2008; Yang et al., 2017; Wang J. et al., 2019; Wang L. et al., 2019). Its unique presence in ConvD mice larynx could possibly be associated with the alleviation of upper respiratory tract infection as reported previously (Meng et al., 2016).

The implementation of artificial introduction of microbiota for the upper airway is a great implication for future translational experiments targeting host–microbe interactions not only within healthy systems, but also within pathological tissues at the level of the vocal folds. Compared to the human oropharyngeal microbiome, the results obtained in our study reflect a remarkable similarity at the phylum level. Human larynx is dominated by Proteobacteria, Firmicutes, Bacteroidetes, Actinobacteria, and Fusobacteria (Huttenhower et al., 2012; Hanshew et al., 2014). Our phyla representation obtained from the mouse samples, including ConvR and ConvD mice groups, resembles that in human larynx, except the absence of Fusobacteria. Additional parallels are apparent when comparing between human oral, laryngeal, and pharyngeal samples and mouse samples at the OTU/ASV and genus levels. Given that we are comparing between human subjects and controlled laboratory mice, the remaining community differences identified are likely due to host-species differences as well as differences in diet and environmental exposures.

In sum, establishing a laryngeal model in which host–microbe interactions can be investigated offers profound potential for advancing our understanding of laryngeal biology. Our results revealed the selective nature of the larynx and provided evidence that gut bacteria can assemble into communities that resemble the commensal residents in ConvR animals phylogenetically and functionally. The successful colonization of laryngeal and oropharyngeal regions with gut microbiota in our study may greatly facilitate the investigation of potential localized inflammatory responses within host tissues that contribute to the disorders of essential laryngeal functions. The evaluation of differences in microbial community composition for regions along the upper airway and upper gastrointestinal tract in a gnotobiotic mouse model enabled us to detect potential nuances in microbial composition along the intersecting gastrointestinal and respiratory tracts. Utilizing said gnotobiotic model to conduct future studies will allow for novel insights into direct microbial contributions to laryngeal epithelial health and pathogenesis. In turn, this presents newfound opportunities to develop and redefine our current treatment paradigms for laryngeal diseases and associated functional disorders.

## Data Availability Statement

The datasets presented in this study can be found in online repositories. The names of the repository/repositories and accession number(s) can be found below: https://www.ncbi.nlm.nih.gov/, BioProject ID PRJNA656992.

## Ethics Statement

The animal study was reviewed and approved by IACUC– University of Wisconsin–Madison.

## Author Contributions

RA, ST, and FR conceived the experiment, analyzed the results, and drafted the manuscript. RA and MG performed sample collection. RA and MG conducted the experiment. All authors reviewed the manuscript.

## Conflict of Interest

The authors declare that the research was conducted in the absence of any commercial or financial relationships that could be construed as a potential conflict of interest.

## Footnotes

1 https://qiime2.org/

2 http://bioinformatics.psb.ugent.be/webtools/Venn/

3 http://bugbase.cs.umn.edu

## Supplementary Material

Supplementary Figure 1 Metagenome functional profiles predicted by PICRUSt2 based on the 16S rRNA gene data.

Supplementary Figure 2 Relative abundance of microorganisms in the laryngeal and fecal microbiota predicted to possess potential phenotypes predicted by BugBase at the organism level. Each dot represents the relative abundance of microorganisms with potential phenotype in one individual sample; lines indicate mean ± standard deviation (SD). Kruskal–Wallis test was applied at the level *P* = 0.05 for the group comparisons (*F* = 36.5).

Supplementary Figure 3 Alpha-rarefaction curves for samples across regions for ConvR and ConvD mice. **(A)** Base of tongue (BOT); **(B)** Esophagus (ESO); **(C)** Larynx (LAR); **(D)** Trachea (TRA). Analysis was performed on a randomly selected subset of 5000 sequences per sample, while sampling depth was set to 3000 for subsequent analysis.

Supplementary Figure 4 Shared ASV between regions in ConvR and ConvD mouse groups, and between mouse groups in each region.

Supplementary Figure 5 Shared ASVs between ConvD and ConvR mice. A total of 465 ASVs were identified across the regions in ConvD and ConvR mice.
