# Supplementary figures and images for: Selective Bacterial Colonization of the Murine Larynx in a Gnotobiotic Model
Source: Front Microbiol. 2020 Nov 4;11:594617. doi: 10.3389/fmicb.2020.594617 (PMC7676279; doi:10.3389/fmicb.2020.594617)

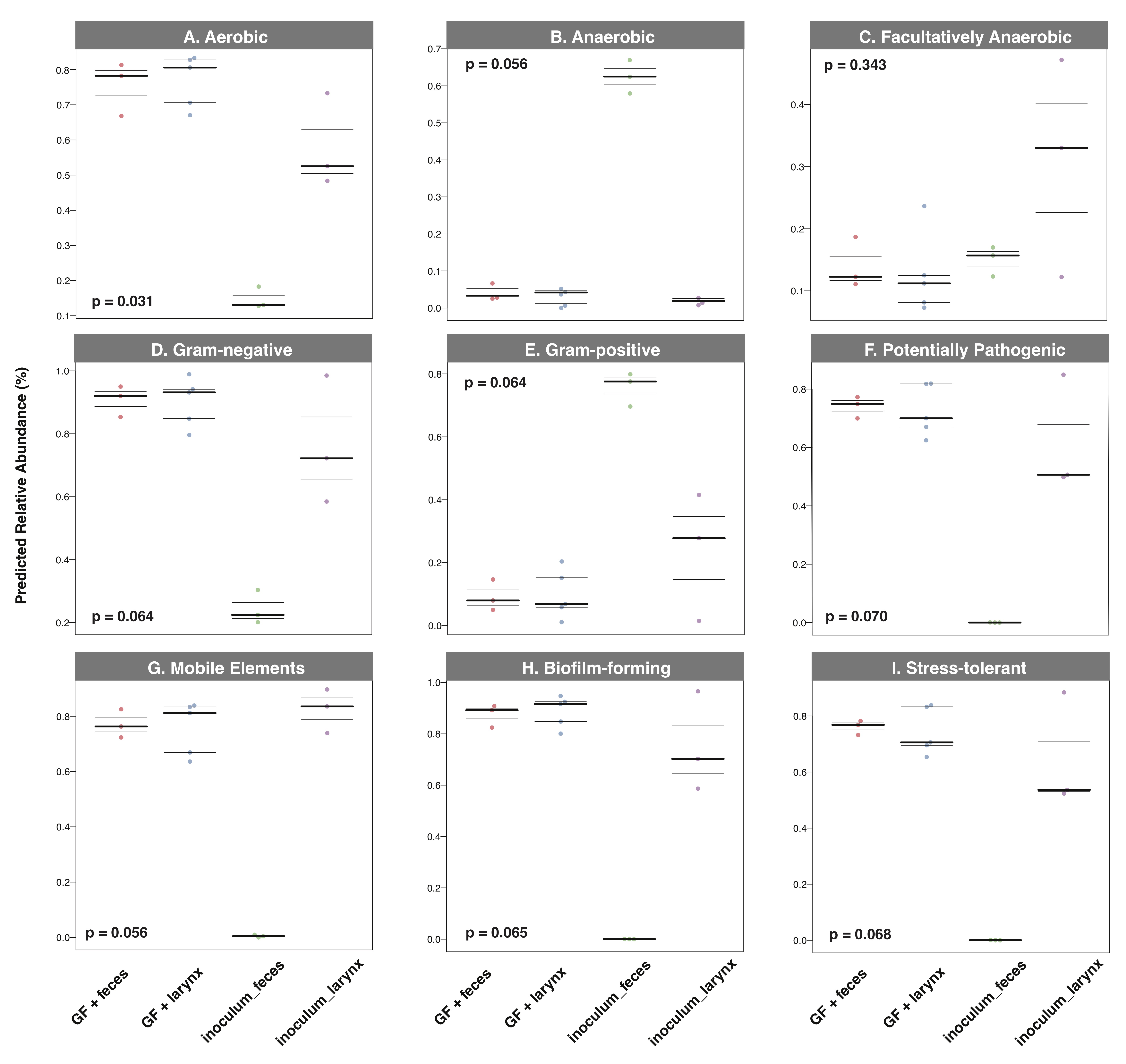

Supplement: Supplementary Figure 1 — Metagenome functional profiles predicted by PICRUSt2 based on the 16S rRNA gene data. [file Image_1.JPEG]

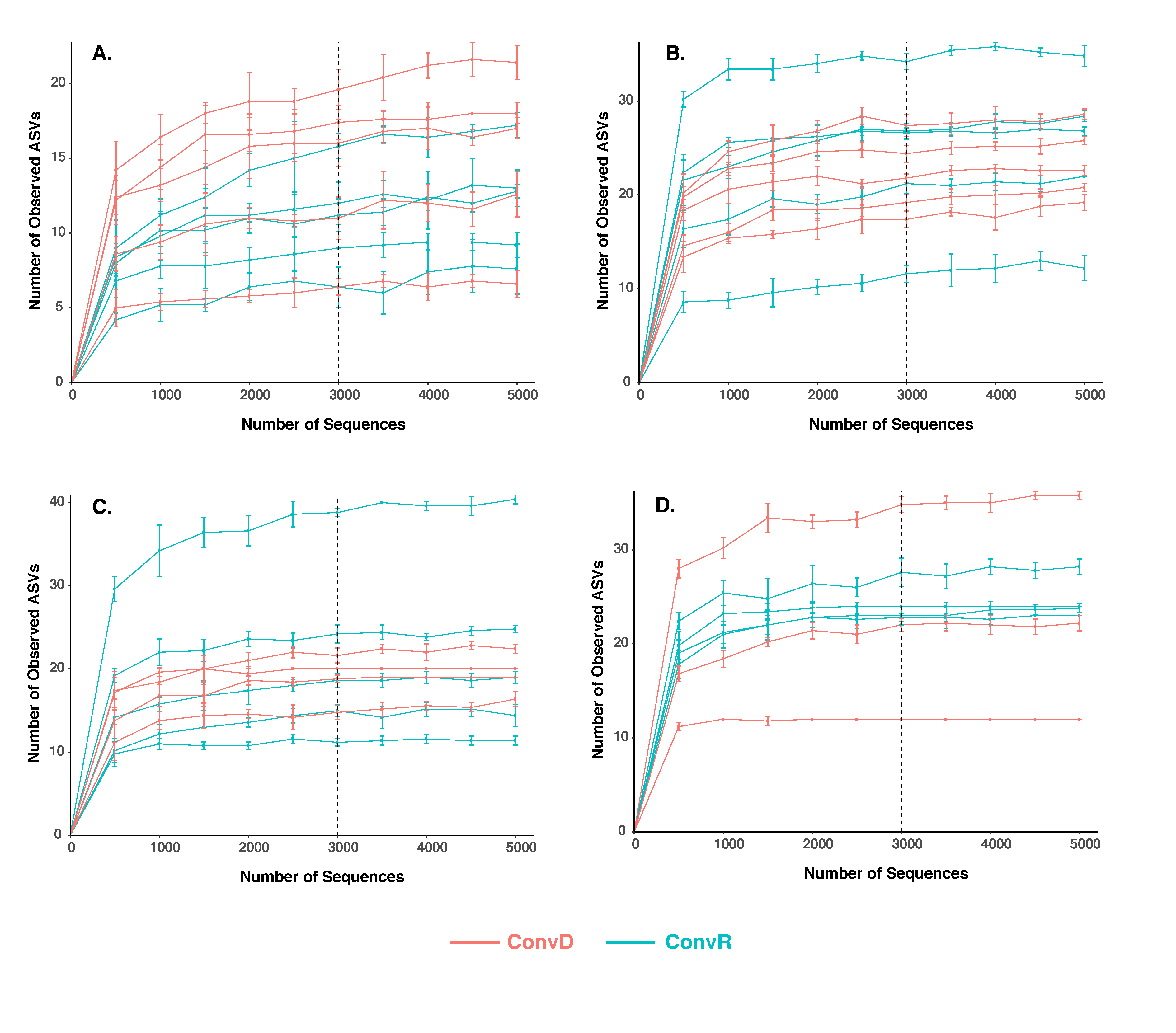

Supplement: Supplementary Figure 2 — Relative abundance of microorganisms in the laryngeal and fecal microbiota predicted to possess potential phenotypes predicted by BugBase at the organism level. Each dot represents the relative abundance of microorganisms with potential phenotype in one individual sample; lines indicate mean ± standard deviation (SD). Kruskal–Wallis test was applied at the level P = 0.05 for the group comparisons (F = 36.5). [file Image_2.TIFF]

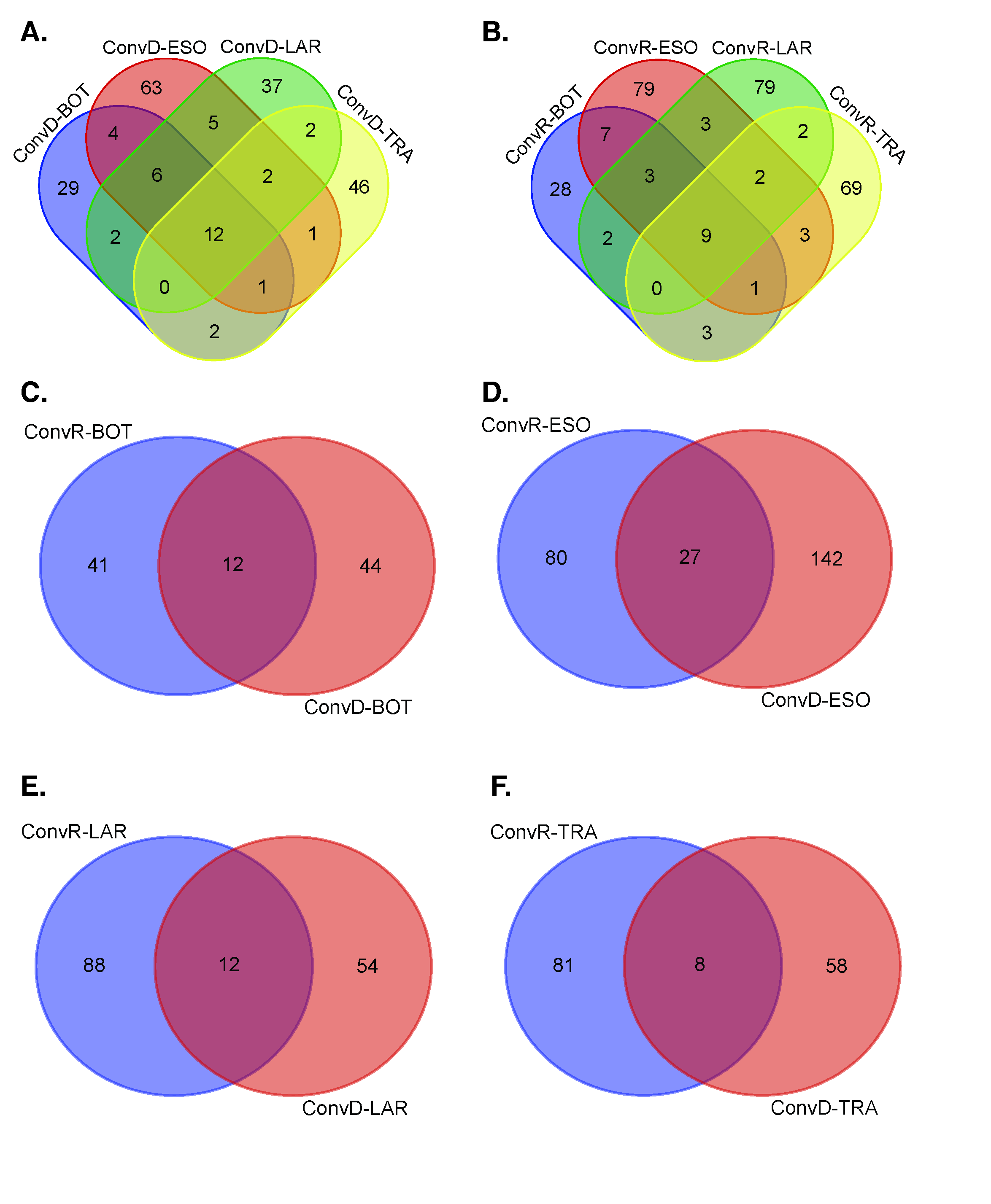

Supplement: Supplementary Figure 3 — Alpha-rarefaction curves for samples across regions for ConvR and ConvD mice. (A) Base of tongue (BOT); (B) Esophagus (ESO); (C) Larynx (LAR); (D) Trachea (TRA). Analysis was performed on a randomly selected subset of 5000 sequences per sample, while sampling depth was set to 3000 for subsequent analysis. [file Image_3.TIFF]

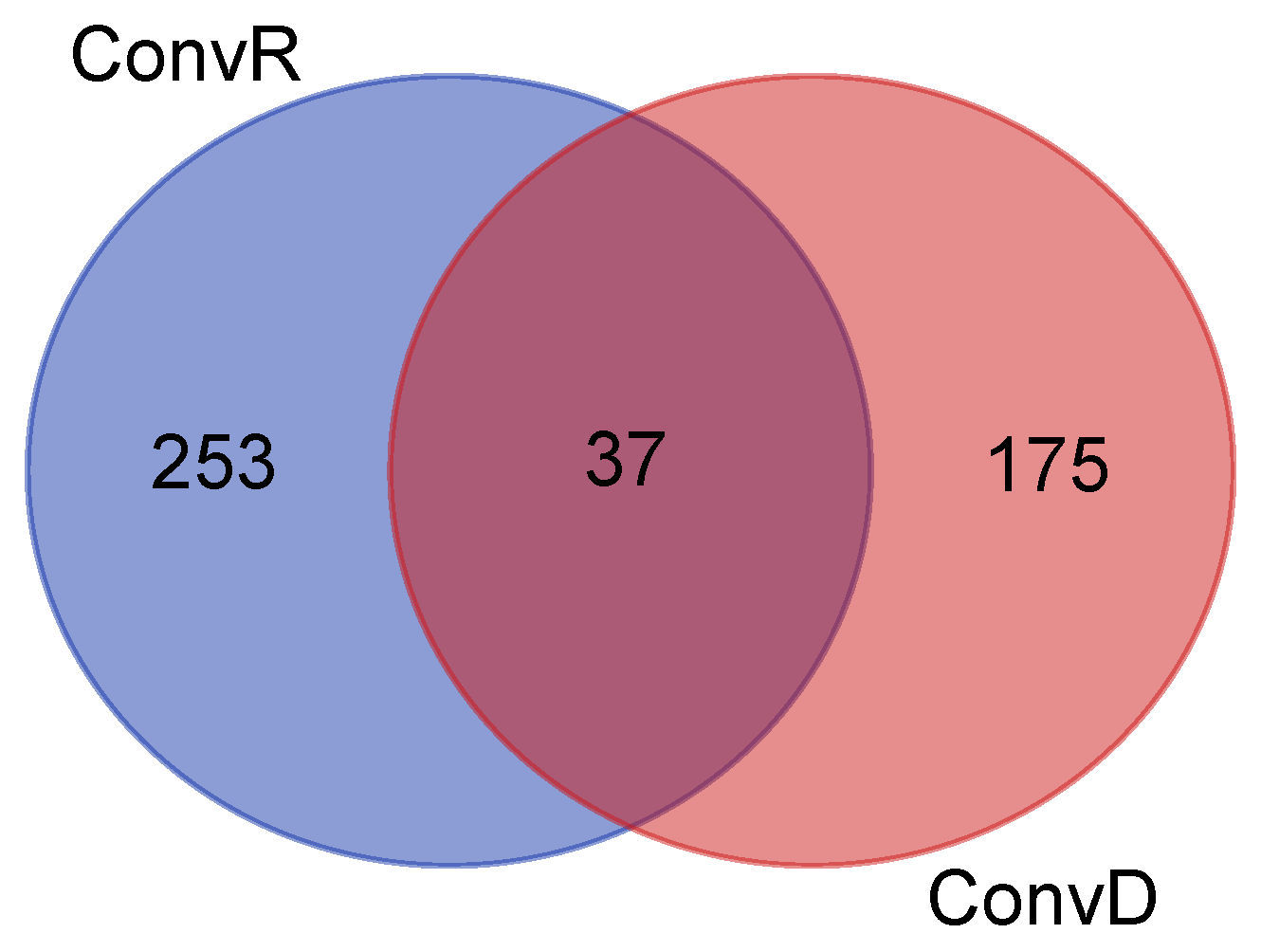

Supplement: Supplementary Figure 4 — Shared ASV between regions in ConvR and ConvD mouse groups, and between mouse groups in each region. [file Image_4.TIFF]

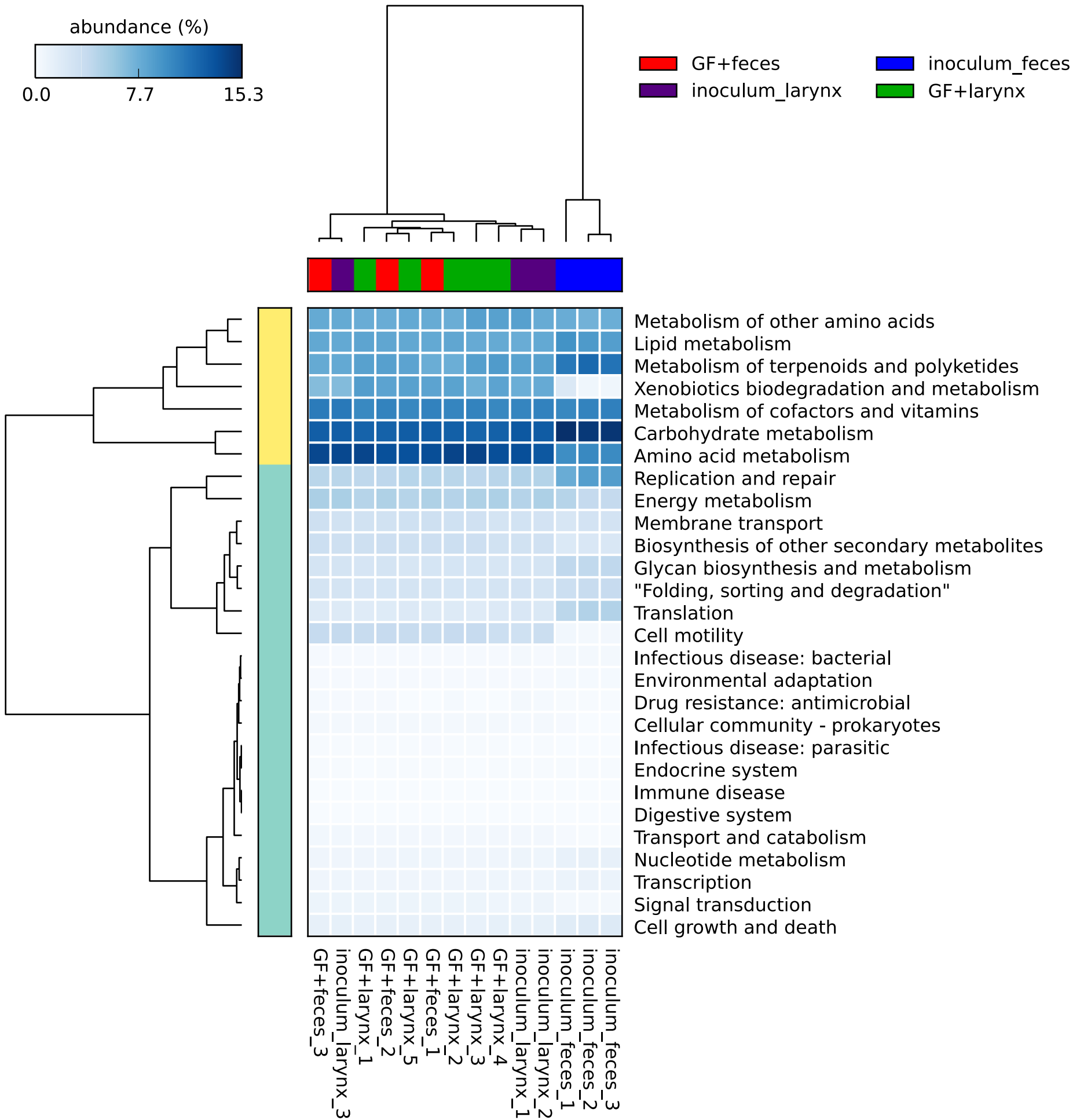

Supplement: Supplementary Figure 5 — Shared ASVs between ConvD and ConvR mice. A total of 465 ASVs were identified across the regions in ConvD and ConvR mice. [file Data_Sheet_1.PDF]
